# Yeast Sensors for Novel Drugs: Chloroquine and Others Revealed

**DOI:** 10.3390/s121013058

**Published:** 2012-09-26

**Authors:** Chantel Swart, Andries Olivier, Khumisho Dithebe, Carolina Pohl, Pieter van Wyk, Hendrik Swart, Elizabeth Coetsee, Lodewyk Kock

**Affiliations:** 1 UNESCO MIRCEN: Microbial, Biochemical and Food Biotechnology, University of the Free State, P.O. Box 339, Bloemfontein 9300, South Africa; E-Mails: swartcw@ufs.ac.za (C.S.); olivieraps@ufs.ac.za (A.O.); dithebek@ufs.ac.za (K.D.); pohlch@ufs.ac.za (C.P.); 2 Centre for Microscopy, University of the Free State, P.O. Box 339, Bloemfontein 9300, South Africa; E-Mail: vanwykpw@ufs.ac.za; 3 Department of Physics, University of the Free State, P.O. Box 339, Bloemfontein 9300, South Africa; E-Mails: swarthc@ufs.ac.za (H.S.); coetseee@ufs.ac.za (E.C.)

**Keywords:** anticancer, anti-malarial, anti-mitochondrial, chloroquine, *Lipomyces*, NanoSAM, preservation, pro-mitochondrial, sensors, yeasts

## Abstract

In this study the mitochondrion is regarded as a target to reveal compounds that may be used to combat various diseases. Consequently, the sexual structures of yeasts (with high mitochondrial activity) were identified as sensors to screen for various anti-mitochondrial drugs that may be toxic to humans and that are directed, amongst others, against fungal diseases and cancer. Strikingly, these sensors indicated that chloroquine is a potent pro-mitochondrial drug which stimulated yeast sexual reproduction. In addition, these sensors also showed that some Non-Steroidal Anti-Inflammatory drugs (NSAIDs), anti-malarial drugs, antifungal and anticancer drugs are anti-mitochondrial. These yeast sensor bio-assays may fast track studies aimed at discovering new drugs as well as their mechanisms and should now be further evaluated for selectivity towards anti-/ pro-mitochondrials, fertility drugs and contraceptives, using *in vitro*, *in vivo*, *in silico* and omics research.

## 1. Introduction

Novel targets and drugs are constantly being sought to effectively combat fungal infections and diseases such as cancer. In this study, we regard the mitochondrion as such a target. Yeast bio-assays, with the sexual structures (asci and ascospores) serving as sensors, have been developed to select new anti-mitochondrial antifungal and anticancer drugs as well as drugs that may adversely affect human mitochondria and consequently health [1–4]. These bio-assays are derived from the so-called Anti-mitochondrial Antifungal Hypothesis. This hypothesis was formulated by Kock and co-workers in 2007 [1] and established a link between: (i) yeast sexual reproduction, (ii) the production of 3-hydroxy (3-OH) oxylipins (via mitochondrial β-oxidation), (iii) mitochondrial activity and (iv) sensitivity towards anti-mitochondrial drugs. For a synopsis of the development of this field of research, which includes yeast sensor smart mechanics linked to Auger nanotechnology, the reader is referred to the video lectures presented by the authors in *e*-conference format [5,6].

According to the Anti-mitochondrial Antifungal Hypothesis, yeasts are divided into two groups: (i) strictly aerobically respiring fungi and (ii) fungi that can obtain energy through aerobic respiration as well as fermentation. As the concentration of anti-mitochondrial drugs added to the yeasts increases, a decrease in the mitochondrial activity and 3-OH oxylipin production in both groups of organisms are expected. The aerobically respiring yeasts should be more susceptible to high anti-mitochondrial drug concentrations than the group that can make use of fermentation as well. The hypothesis also indicates that the sexual stages of both groups of yeasts should be more sensitive towards anti-mitochondrial drugs than the asexual stages. Furthermore, decreased levels of 3-OH oxylipins as well as mitochondrial activity are suggested in asexual cells when compared to sexual cells in both groups.

This hypothesis was visualized by the establishment of yeast bio-assays using the sensing yeasts *Eremothecium ashbyi* and *Nadsonia fulvescens*, mimicking the effects expected from the hypothesis [4]. These yeasts can both ferment and aerobically respire and should therefore be less sensitive to anti-mitochondrial drugs compared to yeasts that can only aerobically respire. Consequently, these bio-assays may not be sensitive enough to detect anti-mitochondrial compounds with low activity, especially since only relatively high drug concentrations could be detected by these bio-assays [4,5].

Therefore the main aims of this study were: (i) to develop a more sensitive anti-mitochondrial yeast bio-assay by using a freshly isolated *Lipomyces* strain that can only respire aerobically; (ii) to screen various compounds as well as preservation by sub-culturing and oxygen limitation with this new bio-assay, (iii) to compare screening results obtained from the *Lipomyces* bio-assay with that of *Eremothecium* and *Nadsonia* and (iv) to also include the well-studied non-fermenting, aerobically respiring yeast *Dipodascopsis* [1,5] as a bio-assay to sense the mitochondrial activity of the anti-malarial drug, chloroquine.

This method of drug screening is a holistic approach that is advantageous compared to specialised *in vitro* screening methods since it takes the cell as a whole (from an intra-omics perspective) into consideration. Yeast mitochondria are similar to mammalian mitochondria and can therefore act as a mirror for the effect of drugs on mammalian mitochondria (especially mitochondrial liabilities posed by new drugs that are occasionally not detected in clinical trials [4]).

## 2. Experimental Section

Experiments were performed at least in triplicate.

### 2.1. Lipomyces: Strain Used, Cultivation and Preservation

*Lipomyces yamadae* (UOFS Y-2824), preserved in the UNESCO MIRCEN culture collection at the University of the Free State, South Africa, was used in this study. Directly after isolation and again after one year of preservation through continuous sub-culturing (every three months) at 25 °C on yeast malt (YM) agar slants [7], *L. yamadae* was streaked out on YM agar plates and incubated at 25 °C to obtain growth for bio-assay experiments.

### 2.2. Lipomyces: Bio-Assay Preparation

*Lipomyces yamadae* was suspended in sterile distilled water (dH_2_O), whereafter 200 μL of the suspension was spread out on YM agar plates. Central wells were made in the plates with sterile Pasteur pipettes. To these wells 46 μL of either 4% or 8% (m/v) acetylsalicylic acid (ASA; Sigma-Aldrich, Steinheim, Germany) dissolved in 96% ethanol (EtOH; Merck, Darmstadt, Germany) were added. An EtOH control was included [2]. All plates were incubated for 11 days at 25 °C. The same technique was used to prepare the *L. yamadae* bio-assay after one year of preservation.

### 2.3. Lipomyces: Confocal Laser Scanning Microscopy (CLSM)

To evaluate whether the sensors of the yeast *L. yamadae* are associated with increased mitochondrial transmembrane potential (Δψ_m_), cells of this organism (grown for 11 days at 25 °C on YM agar plates), were washed with Phosphate Buffered Saline (PBS; Oxoid, Hampshire, England) and treated with 31 μL of the mitochondrial stain Rhodamine 123 (Rh123; Molecular Probes, Invitrogen Detection Technologies, Eugene, OR, USA). These were incubated for 1 h in the dark at 21 °C, washed with PBS and subsequently fixed in Dabco (Sigma-Aldrich) on microscope slides. The cells were then viewed with CLSM (Nikon TE 2000, Tokyo, Japan) to visualize Δψ_m_ [8]. Green fluorescence indicates areas of increased Δψ_m_ and therefore mitochondrial activity.

### 2.4. Lipomyces: Effect of Preservation and Chloroquine on Sensor Development

The protocol included the preparation of bio-assay plates treated with EtOH alone (Control) and chloroquine dissolved in EtOH respectively, all prepared according to Section 2.2. Here ASA was replaced with 8% (m/v) Chloroquine diphosphate (Sigma-Aldrich) in EtOH. After incubation, samples were drawn from two areas of each plate. Each area was sampled at five different points spaced equally across the specific area tested on the plates. Each experiment was performed on separate bio-assay plates in triplicate. A total of 10 fields (40× magnification) was analyzed per sample point and the percentage sensors (*i.e*., asci with ascospores) relative to the total number of asexual cells plus sensors of *L. yamadae* was calculated using a Light Microscope (Axioplan, Zeiss, Göttingen, Germany) coupled to a Colourview Soft Digital Imaging System (Münster, Germany). Therefore, a total of 1 (area) × 5 (sample points in each area) × 10 (fields per sample point) × 3 (triplicate) = 150 fields were analysed per area to obtain a representative relative sensor count. This experiment was repeated after one year of preservation to determine the effects of storage and sub-culturing on sensor development.

### 2.5. Lipomyces: Scanning Electron Microscopy (SEM)

Scanning Electron Microscopy was performed according to the method of Swart and co-workers [9]. Cells from different zones of the plate were chemically fixed with 3% (v/v) glutardialdehyde (Merck) in a sodium phosphate buffer for 3 h. The suspension was rinsed with buffer followed by fixation with 1% (m/v) osmium tetroxide (Merck), also in sodium phosphate buffer for 90 min. The suspension was again washed with buffer and dehydrated in a graded series of EtOH: 30%, 50%, 70% and 90% for 30 min each, with centrifugation between each step, and 2 × 100% EtOH for 90 min each, with centrifugation between each step. The dehydrated yeast cells were submitted to critical point drying, mounted on stubs and coated with a layer of gold—making it electrically conductive. These preparations were viewed with SEM (Shimadzu SSX-550 Superscan, Tokyo, Japan).

### 2.6. Lipomyces: Nano Scanning Auger Microscopy (NanoSAM) and Argon (Ar^+^) Etching

This was performed according to the method of Swart and co-workers [9]. Cells from the dark brown zone of the *Lipomyces* bio-assay were first prepared for SEM observation according to the protocol in Section 2.5 and then subjected to NanoSAM Ar^+^ etching. Here, a PHI 700 Nanoprobe (Chigasaki, Japan) with SAM and SEM facilities was used. The field emission electron gun employed for SEM and SAM analyses was set at 2.45 A filament current, 4 kV extractor voltage and 238.1 μA extractor current, thereby obtaining a 20 kV, 10 nA electron beam for the Auger analyses and SEM imaging. The diameter of the electron beam was 12 nm. The electron gun unit's upper pressure was 8.8E-10 Torr and that in the main chamber was 2.29E-10 Torr. All measurements were performed using aperture A. An 8 μm Field of View (FOV) was used for SEM and the number of frames used was 4. The Auger point analyses were acquired by using 10 cycles per survey, 1 eV per step and 20 ms per step. The Nanoprobe was equipped with an Ar^+^ ion sputtering gun that operated at a beam voltage of 2 kV, an ion beam current of 2 μA and a raster area of 1 × 1 mm. This gave a sputter rate of about 27 nm/min. The ion emission current operated at 15 mA. Finally, an alternating sputter mode with 1 min sputter intervals and 2 min sputter time was used with no rotation.

### 2.7. Lipomyces: Screening of Compounds (Table 1)

Bio-assays were prepared as described in Section 2.2. The disappearance of pigmentation on bio-assay plates was regarded as a positive hit, *i.e.*, selective inhibition of sensor development. This was verified by Light Microscope observation. Various compounds were screened for anti-mitochondrial activity (Table 1). These included Non-Steroidal Anti-Inflammatory drugs (NSAIDs) [46 μL of an 8% (m/v) solution dissolved in 96% EtOH] (Sigma-Aldrich), anti-malarial drugs [46 μL of an 8% (m/v) solution dissolved in 96% EtOH] (Sigma-Aldrich), antifungal drugs (added as E-Test strips) (Davies Diagnostics, Randburg, South Africa), anticancer drugs [46 μL of an 8% (m/v) solution dissolved in 96% EtOH] (Sigma-Aldrich) and three classic respiratory inhibitors (Sigma-Aldrich). The effect of oxygen limitation was also investigated according to the method of Ncango and co-workers [10].

### 2.8. Eremothecium: Strain Used and Cultivation

*Eremothecium ashbyi* (UOFS Y-630), preserved in the UNESCO MIRCEN culture collection at the University of the Free State, South Africa, was cultivated as described in Section 2.1.

### 2.9. Eremothecium: Bio-assay Preparation and Screening of Compounds

The same protocol described in Section 2.2 was used to construct the *Eremothecium* bio-assay [11]. Only an 8% (m/v) in EtOH solution [not 4 % (m/v) EtOH solution as in *Lipomyces*] of the tested compounds was added while the plates were incubated under continuous inspection for pigmentation for 48 h at 25 °C. The screening of compounds was according to Table 1. Results for oxygen limitation and other compounds indicated by an asterisk in Table 1 were obtained from [2,5].

### 2.10. Eremothecium: Confocal Laser Scanning Microscopy (CLSM) and Light Microscopy (LM)

*CLSM:* Cells from the yellow zone were mounted on a microscope slide whereafter one drop of Calcofluor White Stain (Fluka, Buchs, Switzerland) and one drop of 10% potassium hydroxide (Merck) were added. The specimen was covered with a cover slip and after 1 min the slide was examined with CLSM (Nikon TE 2000) using ultraviolet (UV) light at 407 nm.

*LM:* The same cells as above were studied using a Light Microscope (Axioplan, Zeiss, Göttingen, Germany) coupled to a Colourview Soft Digital Imaging System (Münster, Germany).

### 2.11. Eremothecium: Scanning Electron Microscopy (SEM) and Nano Scanning Auger Microscopy (NanoSAM)

*SEM:* This was performed as described in Section 2.5 on yeast cells from different zones of the plate.

*NanoSAM:* Cells, including ascospores from the yellow zone of the *Eremothecium* bio-assay were first prepared for SEM observation according to the protocol in Section 2.5 and then subjected to NanoSAM Ar^+^ etching as described in Section 2.6.

### 2.12. Dipodascopsis: Strain Used and Cultivation

*Dipodascopsis uninucleata* var. *uninucleata* (UOFS Y-0128), preserved in the UNESCO MIRCEN culture collection at the University of the Free State, South Africa, was cultivated as described in Section 2.1.

### 2.13. Dipodascopsis: Bio-Assay Preparation and Effect of Chloroquine on Sexual Stage

The same protocol used to construct the bio-assay of *L. yamadae* (Section 2.2) was used except that only an 8% (m/v) solution of chloroquine in EtOH was added and compared to an EtOH control. The bio-assay plates were incubated for 72 h at 25 °C. After incubation, samples were drawn from three areas (1-3) of each plate as depicted in Figure 4(a,b). Each area was sampled at three points spaced equally across the specific area tested on the plate. Experiments were performed in triplicate on EtOH (Control = Contr.) and chloroquine in EtOH (CQ) bio-assays. A total of 25 fields in each area point were analyzed by using a haemacytometer (40 × magnification) and Light Microscope (Axioplan, Zeiss) coupled to a Colourview Soft Digital Imaging System. This was performed by counting the number of empty sensors (ascospores discharged) as well as sensors filled with ascospores. Therefore, a total of 1 (area) × 3 (sample points in each area) × 25 (fields per sample point) × 3 (triplicate) = 225 fields were analysed per area to obtain a representative relative empty sensor count that depicts percentage discharged sensors.

### 2.14. Dipodascopsis: Confocal Laser Scanning Microscopy (CLSM) of Ascospore Filled Sensor

One drop of a 1% (m/v) Orange G (Molecular Probes) was added to cells with ascospore filled sensors mounted on a microscope slide and incubated for 5 min at 21 °C to allow the stain to penetrate the cells. The stained cells were then examined using a Nikon TE 2000 CLSM.

### 2.15. Dipodascopsis: Nano Scanning Auger Microscopy (NanoSAM) and Ar^+^ Etching

Cells, including ascospores from zone 3 of the *Dipodascopsis* bio-assay were first prepared for SEM observation according to the protocol in Section 2.5 and then subjected to NanoSAM Ar^+^ etching as described in Section 2.6.

### 2.16. Nadsonia fulvescens: Strain Used and Cultivation

*Nadsonia fulvescens* (UOFS Y-0705), preserved in the UNESCO MIRCEN culture collection at the University of the Free State, South Africa, was cultivated as mentioned in Section 2.1 except that the incubation temperature was 21 °C.

### 2.17. Nadsonia fulvescens: Bio-assay Preparation and Screening of Compounds

The same protocols for bio-assay construction and screening described in Sections 2.2 and 2.7 respectively, were followed except that only an 8% m/v in EtOH solution of the tested compounds was added. The compounds screened are listed in Table 1. The plates were incubated for 7 days at 21 °C to observe sensor response. Results for compounds indicated by a double asterisk in Table 1 were obtained from [8].

## 3. Results and Discussion

### 3.1. Yeasts Sensing Anti-Mitochondrial Drugs

**Figure 1.**
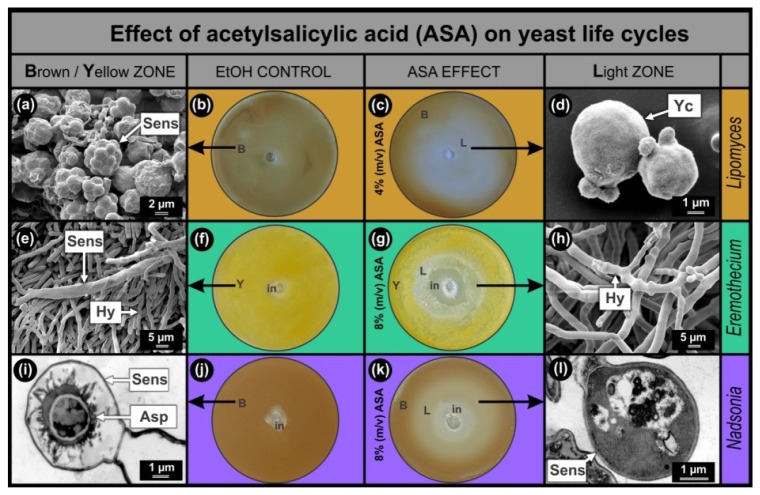
Colour sensors of *Lipomyces yamadae, Eremothecium ashbyi* and *Nadsonia fulvescens* sensing ASA. (**a**) SEM micrograph of sensors with ascospores of *L. yamadae* from brown zone in (b). Brown zone in (c) yielded similar results. (**b**) Control agar plate with brown lawn (due to melanin formation) [5]. The same amount of EtOH added as in (c). (**c**) Bio-assay showing inhibitory effect of ASA on brown pigmented sensor formation (L). (**d**) SEM micrograph of yeasts without sensors in L zone showing selective inhibition of sensors. (**e**) SEM micrograph of yellow sensors of *E. ashbyi* obtained from yellow lawn in (f). Yellow zone in (g) yielded similar results. (**f**) Control agar plate with yellow lawn (due to Riboflavin) [5]. Same amount of EtOH added as in (g). (**g**) Bio-assay showing inhibitory effect of ASA on sensor formation (L). (**h**) SEM micrograph of hyphae without sensors in L zone showing selective inhibition of sensors. (**i**) TEM micrograph showing sensor structure and ascospore of *N. fulvescens* observed in brown zone in (j). Brown zone in (k) yielded similar results. (**j**) Control bio-assay with brown lawn (due to melanin) [5,6]. The same amount of EtOH added as in (k). (**k**) Bio-assay showing inhibitory effect of ASA on sensor formation (Light zone-L). (**l**) TEM micrograph of sensor containing a malformed ascospore in L zone showing selective inhibition. Images of *Nadsonia* (i,l) were taken with permission from [8,9]. Parts of this figure are available as a video lecture [5]. ASA, Acetylsalicylic acid; Asp, ascospore; B, brown zone; Hy, hyphae; in, inhibition zone; L, light zone; Sens, sensor; Y, yellow zone; Yc, yeast cell.

**Figure 2.**
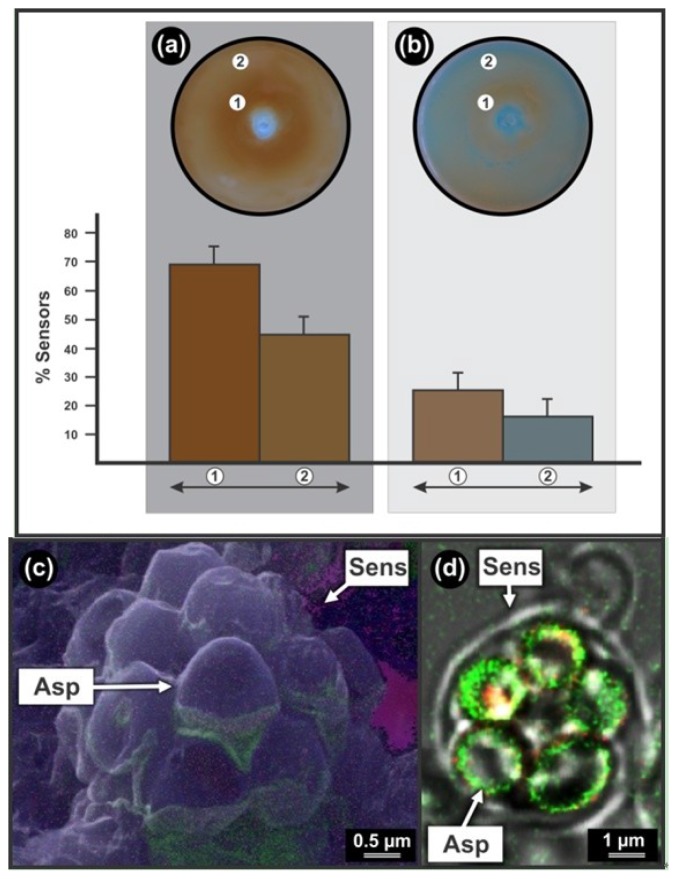
Colour sensors of *Lipomyces yamadae* sensing Chloroquine and the effect of preservation. (**a**) Bio-assay preparation without prior preservation of indicator yeast. Column 1 represents the percentage sensors relative to asexual cells counted in the darker brown zone (indicated as 1). Column 2 indicates the percentage sensors obtained in the lighter brown zone (indicated as 2). (**b**) Bio-assay preparation after one year of preservation. Column 1 indicates the percentage sensors relative to asexual cells counted in the darker brown zone (indicated as 1). Column 2 represents the percentage sensors in the lighter brown zone (indicated as 2). Values are the averages of a least three independent experiments and the bars indicate the standard deviation. (**c**) Sensor disassembly by Argon (Ar^+^) etching visualized by Nano Scanning Auger Microscopy (NanoSAM). Green, gold; Blue, carbon; Pink, oxygen. (**d**) Increased fluorescence (green) surrounding ascospores inside sensor as observed by Confocal Laser Scanning Microscopy (CLSM). Parts of this figure (a,c) are available in a video lecture posted on the website of Translational Biomedicine and are reproduced with permission [5].

Results of the anti-mitochondrial drug ASA, obtained with the *Lipomyces*, *Eremothecium* and *Nadsonia* bio-assays were compared (Figure 1). In all cases, the bio-assay sensors are pigmented which facilitates screening of compounds that stimulate (increase in colour intensity) or inhibit (decrease in colour intensity) sensor development. As was expected from the hypothesis, increased mitochondrial activity was observed in sensors of *Lipomyces* as well as *Eremothecium* [2] and *Nadsonia* [8] (Figure 2(d)). This increase was mainly associated with the ascospores inside the sensors. In all bio-assays studied, the EtOH controls developed homogeneously pigmented lawns, *Lipomyces*–brown (Figure 1(b)); *Eremothecium*–yellow (Figure 1(f)) and *Nadsonia*–brown (Figure 1(j)). These lawns contained matured pigmented sensors (Figure 1(a,e,i)) as observed by SEM (Figure 1(a,e)) and Transmission Electron Microscopy (TEM) (Figure 1(i)). When the anti-mitochondrial ASA was added, all bio-assays developed light zones (L) where pigmentation and hence sensor development were inhibited (Figure 1(c,g,k)). Ultra-structural imaging showed that sensors were absent or malformed in these zones (Figure 1(d,h,l)) and that ASA selectively inhibited these sensors at sub-lethal concentrations (Figure 1(g,k)).

As expected, we found that the *Lipomyces* bio-assay was more than twice as sensitive to ASA compared to the other two bio-assays. A larger sensor inhibition zone (Figure 1(c)) in the *Lipomyces* assay was observed, when 4% (m/v) ASA solution was added compared to when an 8% (m/v) ASA solution was added to the other two fermenting yeasts. This was to be expected since *Lipomyces* can only obtain energy from an aerobic respiratory metabolism and not from fermentation as well [5]. It is interesting to note that when *Lipomyces* was grown on 8% (m/v) ASA, still no growth inhibition was observed (results not shown). This means that the asexual cells of *L. yamadae* are more resistant towards this anti-mitochondrial drug compared to *E. ashbyi* and *N. fulvescens*. This cannot be explained at present.

Various compounds with known and unknown anti-mitochondrial activities were screened with the *Lipomyces*, *Eremothecium* and *Nadsonia* bio-assays and compared with reports from the Food and Drug Administration (FDA) on drugs posing mitochondrial liabilities [4,12] (Table 1). No discrepancies between screening results of the three yeast bio-assays could be found.

Compounds posing mitochondrial liabilities according to FDA Black Box Warnings as well as those that gave positive hits in all three bio-assays include diclofenac, ibuprofen and indomethacin. Compounds that tested positive for Black Box Warnings as well as *Lipomyces* and *Eremothecium* bio-assays include naproxen, peroxicam and sulindac. However, diflunisal and fenoprofen tested negative with Black Box Warnings but positive with the bio-assays. Interestingly, these NSAIDs were also reported to be anti-mitochondrial in literature [2,12,13]. The NSAIDs, ASA and salicylic acid tested positive for all three the bio-assays. These results also corroborate previous mammalian and plant studies suggesting that the NSAIDs, ASA, ibuprofen, indomethacin and salicylic acid also have anti-mitochondrial activity [14–17]. The anti-malarial drug quinine tested negative for both *Lipomyces* and *Eremothecium* bio-assays, while chloroquine tested negative for all bio-assays. Artemisinin gave a positive hit when tested with the *Eremothecium* bio-assay. This was to be expected since the anti-mitochondrial activity of this important anti-malarial drug is well reported in yeasts [18]. Thapsigargin, capable of collapsing membrane potential of mitochondria in the malaria parasite [19] also gave a positive hit with the *Eremothecium* bio-assay. In addition, all antifungal agents submitted to the bio-assays tested positive with all three yeast bio-assays (Table 1).

The anti-mitochondrial anticancer drug lonidamine [20] tested positive with the *Lipomyces* and *Eremothecium* bio-assays. In addition, CD 437 and betulinic acid also tested positive with the *Lipomyces* bio-assay. Both of these anticancer drugs are known to have anti-mitochondrial activity [21,22].

The effect of three classic respiratory inhibitors was also investigated in order to serve as benchmark for the efficacy of these bio-assays. All three bio-assays were sensitive to antimycin A and oxygen limitation, while rotenone also tested positive with the *Lipomyces* bio-assay.

### 3.2. Yeasts Sensing Pro-Mitochondrial Drugs

#### 3.2.1. Lipomyces yamadae

##### Sensor Bio-Assay

*Chloroquine effect:* The anti-malarial drug chloroquine had a stimulatory effect on the sensor development in the *Lipomyces* bio-assay (Figure 2(a,b)). With increase in drug concentration (from periphery towards centre, *i.e.*, sub-lethal zone) a drastic increase (P < 0.05; Two tailed t-Test) from 44.3% ± 10.0% (Figure 2(a)-2) to 69.8% ± 9.8% (Figure 2(a)-1) in sensor development was observed. This is visualised by the development of a concomitant darker brown zone closer to the well.

After one year of preservation, a similar trend was experienced. Here, the percentage sensors in the darker brown zone was 25.6% ± 7.6% (Figure 2(b)-1), while the percentage sensors in the lighter brown zone was 16.6% ± 9.1% (Figure 2(b)-2), indicating again a significant (P < 0.05; Two tailed t-Test) stimulatory effect of chloroquine on the sensor development and expected mitochondrial activity after preservation. Strikingly, this stimulatory effect has also been reported in the literature where sub-lethal doses of chloroquine elevate the levels of *Plasmodium* gametocytes (*i.e.*, start of sexual phase) in the host's blood by favoring asexual merozoite transition to sexual gametocytes, thereby ensuring more effective transmission of the parasite via the mosquito to the host. This effect has also been shown to be enhanced in resistant *Plasmodium* strains [23]. It is interesting to note that chloroquine also influences mitochondrial activity by stimulating respiration in bovine spermatozoa [24].

*Preservation effect:* When comparing bio-assays before and after one year of preservation by continuous sub-culturing, it is clear that this preservation method inhibited sensor development (Figure 2(a,b)). The results indicate a 2.7 times significant decrease (from 69.8% ± 9.8% to 25.6% ± 7.6%; P < 0.05; Two tailed t-Test) in the ability to produce sensors in response to chloroquine at sub-lethal concentration over one year of preservation (compare Figure 2(a)-1 to Figure 2(b)-1). Strikingly, the same level of decrease was observed at lower concentrations of chloroquine (compare Figure 2(a)-2 to Figure 2(b)-2) with again a 2.7 times significant decrease in the ability to produce sensors (from 44.3% ± 10.0% to 16.6% ± 9.1%; P < 0.05, Two tailed t-Test). This is also visualised by the concomitant decrease in sensor associated pigmentation. Similar levels of sensor development were recorded when comparing sensor development at lower chloroquine concentrations at the periphery of respective plates (Figure 2(a)-2 and 2(b)-2)) with the periphery of control plates, *i.e.*, when only EtOH was added to bio-assays before and after one year of preservation (results not shown).

The above results suggest that preservation through sub-culturing is also anti-mitochondrial. Interestingly, according to literature [25] sub-culturing over a long period may have mutagenic effects on yeasts leading to changes in morphological characteristics and loss in the ability to sporulate.

This may explain the decrease in pigmented sensor development in the strictly respiring *Lipomyces* over one year of this type of preservation. Preservation by sub-culturing over extended periods can therefore be considered anti-mitochondrial where the sexual structures (*i.e*., sporulation) of yeasts, that can only obtain energy from aerobic respiration, should be the most sensitive.

##### Sensor Structure

Sensors are characterised by various smooth spherical ascospores of about 2 μm in diameter all enveloped by a cell wall (Figure 2(c,d)). This is exposed by sensor disassembly using Ar^+^ etching and NanoSAM in SEM mode (Figure 2(c)). Here Auger nano-probing shows remnants of gold (in green) on sensor wall (from gold sputtering during NanoSAM preparation) as well as element composition of ascospores (*i.e.*, carbon in blue and oxygen in pink). As expected [4] these sensors have increased mitochondrial activity indicated by increased fluorescence associated with ascospores (Figure 2(d)). Here no smart mechanics, as is evident in the other sensing yeasts, were observed.

#### 3.2.2. Eremothecium ashbyi

##### Sensor Bio-Assay

**Figure 3.**
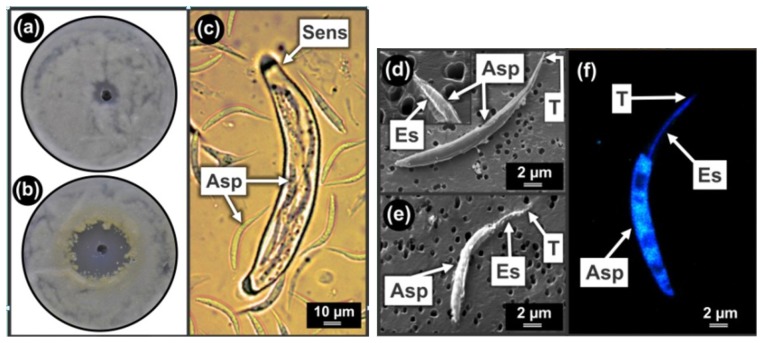
Colour sensors of *Eremothecium ashbyi* sensing Chloroquine. (**a**) Ethanol (EtOH) control plate showing no yellow pigmentation and thus no stimulation of sensor development. (**b**) Bio-assay plate treated with chloroquine dissolved in EtOH showing yellow pigmentation at sub-lethal concentrations that is indicative of sensor development and pro-mitochondrial activity. (**c**) Light Microscope micrograph of a sensor comprised of an ascus filled with sickle shaped ascospores. (**d**) Nano Scanning Auger Microscopy (NanoSAM) in Scanning Electron Microscopy (SEM) mode showing ultra-structure of ascospore. Insert: The endoskeleton exposed inside part of ascospore by nano etching using Argon (Ar^+^) linked to NanoSAM in SEM mode. (**e**) Ar^+^ etched ascospore tip exposing endoskeleton. (**f**) Confocal Laser Scanning Microscopy (CLSM) micrograph showing a chitinous endoskeleton (in blue) inside a sickle shaped ascospore. Asp, ascospore; Es, endoskeleton; Sens, sensor; T, ascospore tip.

Similarly to *L. yamadae*, chloroquine also stimulated pigmented sensor development in the *Eremothecium* bio-assay (Figure 3(a,b)). At sub-lethal concentrations of the drug, yellow pigmentation started to develop next to the inhibition zone (Figure 3(b)). This was not visible in the EtOH control (Figure 3(a)). This is indicative of increased sensor development as well as increased mitochondrial activity [2]. It is important to note that the yellow zone appeared within 24 h after which the whole plate coloured yellow. Therefore, if the plate was inspected after 24 h, no effect of chloroquine would have been observed as was reported in [5].

##### Sensor Structure and Smart Mechanics

In this study, the moving parts of the sensor (*i.e.*, ascospores) were disassembled and further imaged by applying CLSM on Calcofluor treated samples as well as NanoSAM linked to Ar^+^ etching. On the basis of our results, we conclude that each sickle shaped ascospore (Figure 3(c)) contains a chitinous endoskeleton (Figure 3(f)) that stretches across its length (Figure 3(d,e)) thereby probably reinforcing its structure to eventually pierce through the sensor upon release from the ascus. This is the first report of an endoskeleton in yeast.

Smart mechanics inside the sensor is made possible by water movement across V-shaped fins situated on the blunt end of the ascospore. These fins are covered with anti-mitochondrial sensitive, beta-oxidation produced 3-OH oxylipins. However, these fins may be scraped off during release through tight fitting sensor cell walls as depicted in Figure 3(d) [5,26]. Sensor-ascospore smart mechanics can be viewed as a movie in video lecture format published by the Kock-group [5].

#### 3.2.3. Dipodascopsis uninucleata

##### Sensor Bio-Assay

**Figure 4.**
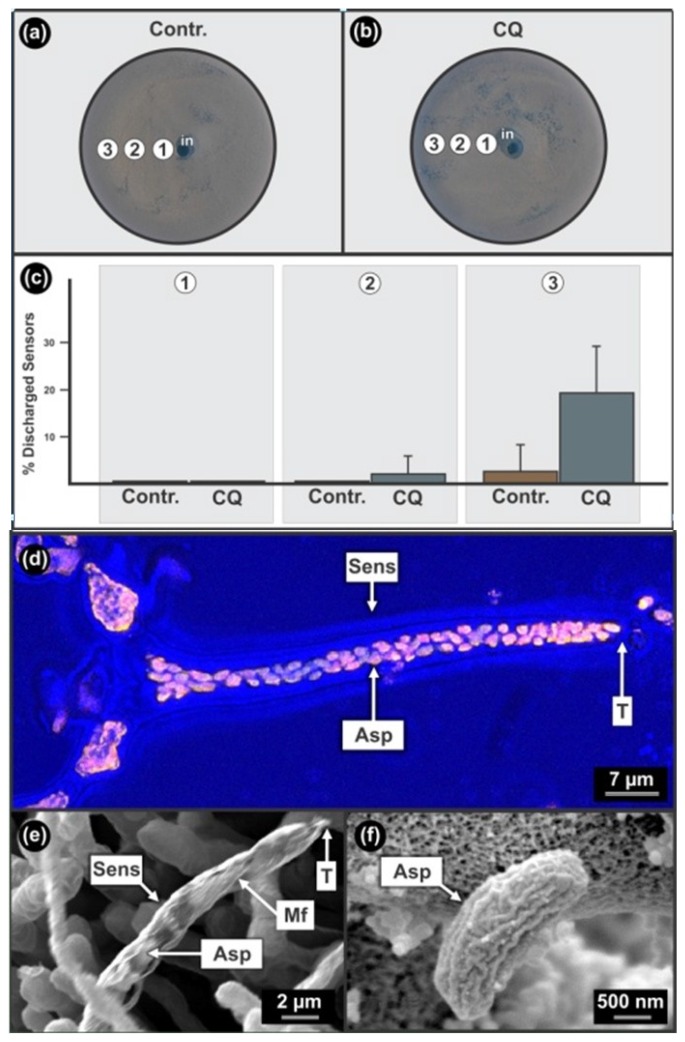
Sensors of *Dipodascopsis uninucleata* sensing Chloroquine by ascospore discharge. (**a**) Control: Bio-assay plate showing three areas (1,2,3) that coincide with a concomitant decrease in concentration of ethanol (EtOH) alone. (**b**) CQ: Bio-assay plate showing three areas (1,2,3) that coincide with a concomitant decrease in the concentration of chloroquine in combination with EtOH. (**c**) A graph showing changes in percentage discharged sensors over a decreasing gradient (from 1 to 3) of EtOH alone (Contr.) and EtOH in combination with chloroquine (CQ). (**d**) A Confocal Laser Scanning Microscope (CLSM) micrograph of an Orange G treated fluorescing sensor of *D. uninucleata.* (**e**) Disassembly of the sensor by Argon (Ar^+^) etching and visualised with Nano Scanning Auger Microscopy (NanoSAM) in SEM mode showing micro-fibrils coiled around the sensor. (**f**) SEM micrograph of an ascospore showing ascospore longitudinal grooves necessary for smart sensor mechanics. Asp, ascospore; Contr., control; CQ, Chloroquine in EtOH solution; Mf, micro-fibrils, Sens, sensor; T, tip of sensor; 1,2,3, zones on bio-assay plates. Part of this figure (*i.e.*, (e)) is available in a video lecture posted on the website of Translational Biomedicine and are reproduced with permission [5].

When chloroquine was added to the bio-assay of *D. uninucleata* (Figure 4(a,b)), we found an inhibition of ascospore discharge from sensors at sub-lethal concentration (Figure 4(c)-1CQ)). However, since the same phenomenon was observed in the EtOH control, this is probably an EtOH effect (Figure 4(a)-1Contr.)). At lower concentrations (Figure 4(c)-2CQ)), this drug enhanced ascospore release compared to samples from a similar position on the control plate (Figure 4(c)-2Contr.)) resulting in increased discharged empty sensors from 0.0% to 2.4% ± 3.8%. When comparing the EtOH control (Figure 4(c)-3Contr.)) to experiments where chloroquine dissolved in EtOH was added (Figure 4(c)-3CQ.)), we found that a significant increase (P < 0.05; Two tailed t-Test) in ascospore discharge from sensors occurred at the periphery of the plates *i.e.*, from 2.9% ± 5.7% to 19.6% ± 9.7%. It is interesting to note that a decrease in the concentration of EtOH in the absence of chloroquine (Figure 4(c)-1 to 3)), also enhanced ascospore release but at a significant lower level compared to when chloroquine was added (P < 0.05; Two tailed t-Test). Unfortunately these sensors are not pigmented and yeast sensing had to be followed by means of time consuming microscopic investigations.

##### Sensor Structure and Smart Mechanics

These sensors are bottle-shaped structures containing coiled micro-fibrils, each with a narrow opening at the tip, allowing the release of only one ascospore at a time, and a broader base containing many ascospores (Figure 4(d,e)). Discharged sensors are formed when elongated grooved ascospores of about 1 μm in diameter (Figure 4(f)) are individually released from sensors (Figure 4(e)) through tight fitting narrow sensor openings of about 1–2 μm in diameter (Figure 4(d)). Ascospore release occurs by smart ascospore rotation mechanics affected by micro-fibril coiled rifling and lubrication by anti-mitochondrial drug sensitive, β-oxidation products such as 3-OH oxylipins [1]. These smart sensor mechanics can be viewed as a movie in video lecture format [5,6,26]. We conclude that since chloroquine had an enhancing effect on ascospore discharge from sensors, it should be pro-mitochondrial, probably also enhancing beta-oxidation at lower concentrations—especially since blocked sensors that were filled with well developed, unreleased ascospores, were formed at higher concentrations of this drug (results not shown). Whether chloroquine has a direct or indirect effect on mitochondrial activity should now be investigated.

## 4. Conclusions

In this study, various compounds including well known mitochondrial inhibitors as well as physical conditions such as preservation by sub-culturing and oxygen limitation, gave positive hits when subjected to the anti-mitochondrial yeast bio-assays. Here mitochondrial activity is probably needed to provide sufficient energy for the complex processes necessary for sensor development. Inhibition of mitochondrial activity may lead to insufficient energy production needed for proper development and pigmentation. Care should however be taken when interpreting these results. Drugs and physical effects that inhibit sensor development may not necessarily influence mitochondrial activity directly. They may, amongst many others, influence parts of the complex synthesis processes involved in sensor development such as meiosis, the formation of secretory vesicles, septin, dityrosine layers, membranes and cell walls as well as pigment production. This may then indirectly lead to the termination of mitochondrial biogenesis and function. It is therefore important that a positive hit obtained with these yeast bio-assays be followed up by further *in vitro*, *in vivo*, *in silico* and omics research to elucidate the inhibitory action of these drugs and physical effects at molecular level [4]. For instance, when a positive hit is encountered, more *in vitro* mechanistic studies can be applied to study the effects of drugs/physical effects on oxidative phosphorylation, permeability transition, ΔΨ_m_ status and other anti-mitochondrial drug targets. This may then be followed up by different tests, using amongst others NanoSAM imaging, enzyme-linked assays of the different respiratory complexes, different histochemical and immuno-histochemical methods as well as animal models to finally predict clinical outcome [4]. We suggest that these bio-assays may find application as a preliminary screening method during lead selection, especially by taking advantage of associated sensor pigmentation.

Of particular interest will be research concerned with the application of NSAIDs and mitocans (anticancer drugs targeting mitochondria of cancer cells) as new anti- mitochondrial anti-fungal drugs and *vice versa*. This approach may even give new careers to conventional drugs used today to treat specific diseases. During the screening for anti-mitochondrial drugs, we unexpectedly uncovered that the anti-malarial drug chloroquine gave opposite results, *i.e.*, stimulating sensor development in bio-assays of *Eremothecium*, *Lipomyces* as well as *Dipodascopsis*. This implicates a pro-mitochondrial action. This finding is of great importance to the field of malarial research and invigoration (increase mitochondrial activity) of cells. This should be followed up with urgency. For many years, chloroquine has been considered the gold standard for treatment of malaria. However resistance build-up by the infective parasite hampers its application. This drug not only targets the asexual stage of the parasite, it also induces gametocytogenesis in the host, a prelude to sexual reproduction occurring in the mosquito vector and a process that renders the vector infective. Chloroquine can therefore also favor malaria infection, an aspect that needs urgent attention [23]. Furthermore, it was reported that chloroquine also increased birth weight during first pregnancy [27]. Here we report yeast bio-sensors that mimics activities of chloroquine in the human host. We found that this anti-malarial drug drastically enhanced the sexual cycle of the yeast *Lipomyces* by more than 63% at sub-lethal concentrations when compared to controls, implicating a strong pro-mitochondrial action.

We conclude that these assays may be used as models to fast track basic studies involved in screening and understanding the mechanism of action of new anti-mitochondrial drugs and possibly even drugs with contraceptive and fertility-promoting action. These sensors may also be used to study physical effects on mitochondrial activity and sexual reproduction such as oxygen limitation as well as preservation.

## Figures and Tables

**Table 1.** Compounds screened with the colour sensors of *Lipomyces*, *Eremothecium* and *Nadsonia*, and compared to the Food and Drug Administration (FDA) Black Box Warnings for mitochondrial liabilities of drugs in use today.

**Compound Tested**	**Selective Inhibition of Sensors**			**Black Box**

	*Lipomyces*	*Eremothecium*	*Nadsonia*	
**NSAIDs**				
ASA	+	+ *	+ **	
Diflunisal	+	+	+	−
Diclofenac	+	+	+	+
Fenoprofen	+	+	Nt	−
Ibuprofen	+	+ *	+ **	+
Indomethacin	+	+ *	+	+
Naproxen	+	+	Nt	+
Peroxicam	+	+	Nt	+
Salicylic acid	+	+ *	+ **	
Sulindac	+	+	Nt	+
**ANTI-MALARIAL DRUGS**				
Artemisinin	Nt	+	Nt	
Chloroquine	-	−	−	
Quinine	-	−	Nt	
Thapsigargin	Nt	+	Nt	
**ANTIFUNGAL DRUGS**				
Caspofungin	+	+	+	
Fluconazole	+	+	+ **	
Itraconazole	+	+	+	
Ketoconazole	+	+	+	
Posaconazole	+	+	+	
Voriconazole	+	+	+	
**ANTICANCER DRUGS**				
Lonidamine	+	+ *	Nt	
CD 437	+	Nt	Nt	
Betulinic acid	+	Nt	Nt	
**CLASSIC RESPIRATORY INHIBITORS**				
Antimycin A	+	+	+	
Oxygen limitation	+	+ *	+	
Rotenone	+	Nt	Nt	

+ = inhibitory; − = no inhibitory effect; Nt = not tested;

* = data taken from [2];

** = data taken from [8]; Black Box Warnings = data taken from [12]. Data taken with permission from a video lecture presented by authors in *e*-conference format [5].
